# Supplementary material for: Model Comparison of Heritability Enrichment Analysis in Livestock Population
Source: Genes (Basel). 2022 Sep 13;13(9):1644. doi: 10.3390/genes13091644 (PMC9498849; doi:10.3390/genes13091644)
Supplement: Supplementary file 1 [file genes-13-01644-s001.zip › genes-1889547-Supplementary.pdf]

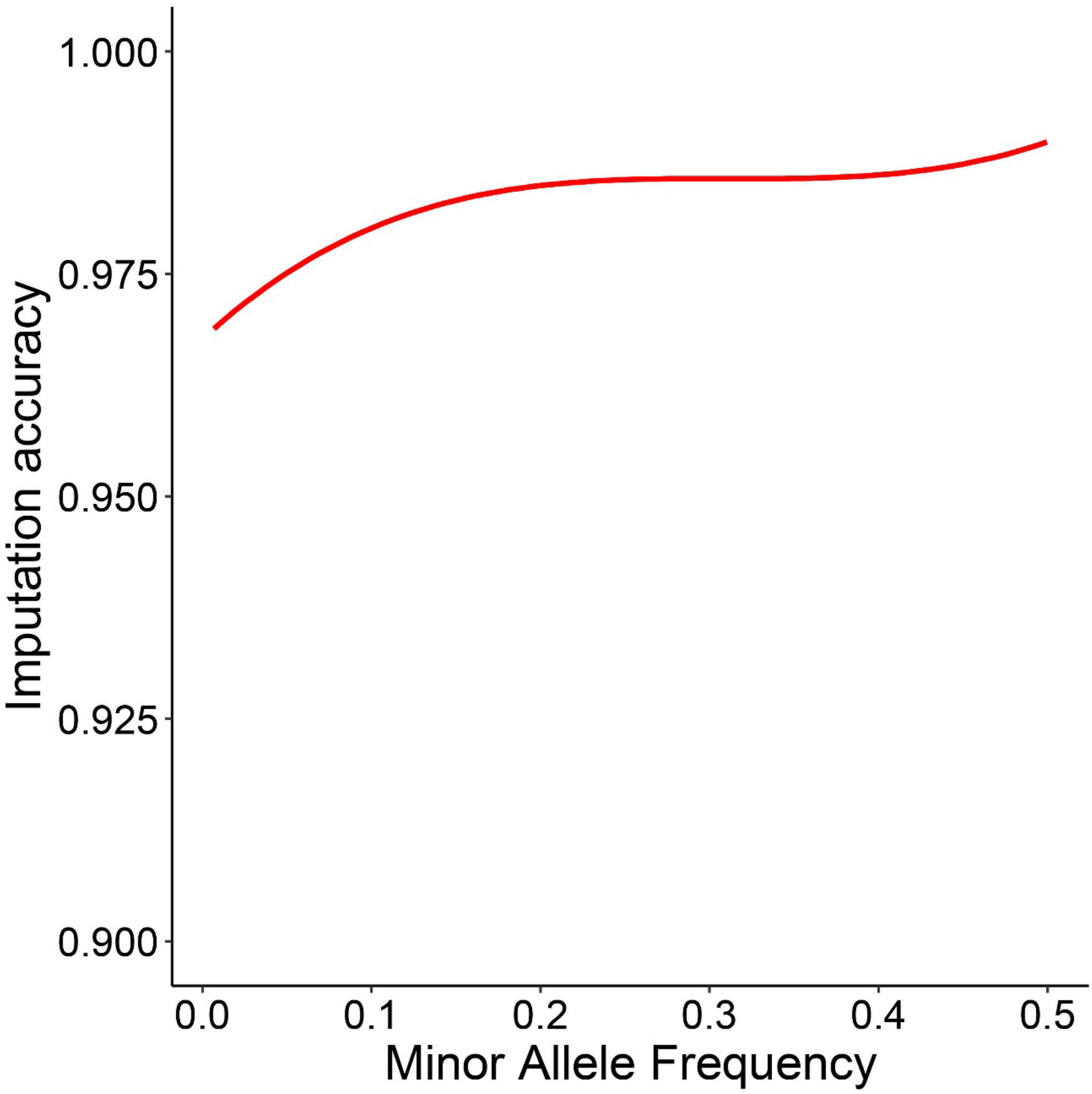

Figure S1. The imputated accuracy of genotype imputation for different minor allele frequency range.

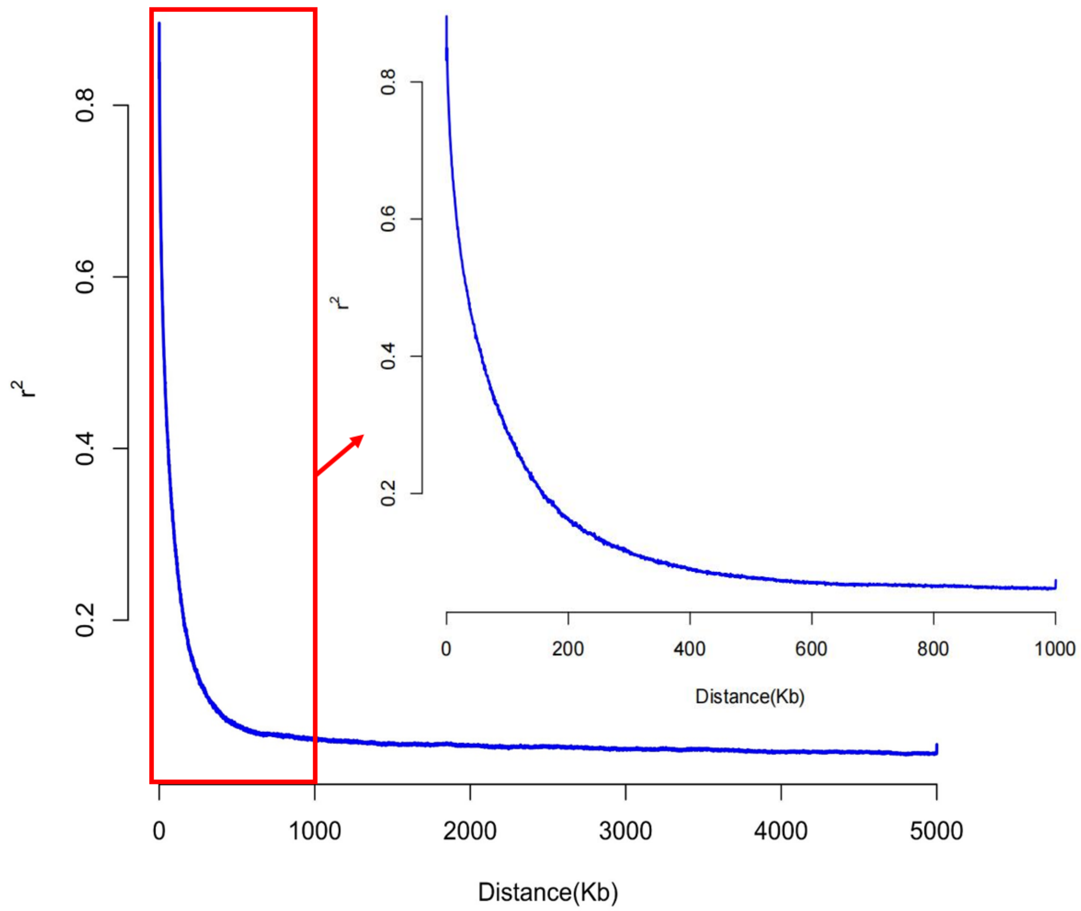

Figure S2. LD decay for the German Holstein population.

Table S1. Models for estimating heritability enrichment.

Model	GRM	Per-SNP heritability
VanRaden model	$\frac{MM^T}{2\sum_1^m p_i(1-p_i)}$	$var(\beta_i) \propto p_i(1-p_i)$
GCTA model	$\frac{MM^T}{\sum_1^m 2p_i(1-p_i)}$	$var(\beta_i) \propto 1$
α-model	$\frac{MM^T}{2\sum_1^m [p_i(1-p_i)]^{1+\alpha}}$	$var(\beta_i) \propto (p_i(1-p_i))^{1+\alpha}$<br>$\alpha$=-1.25、-1、-0.75、-0.5、-0.25、0、0.25
LDAK model	$G=\frac{MWM^T}{2\sum_1^m [p_i(1-p_i)]^{0.75}}$	$var(\beta_i) \propto (p_i(1-p_i))^{0.75} w_i^{*1}$

[1] $w_i^* = w_i \frac{m'}{\sum_i w_i}$

Table S2. Heritability estimation for different models.

Model	Phenotype	h_F^2	h^2
VanRaden model(α=0)	T1	0.246	0.776
VanRaden model(α=0)	T2	0.258	0.794
VanRaden model(α=0)	T3	0.101	0.542
GCTA model(α=-1)	T1	0.263	0.803
GCTA model(α=-1)	T2	0.262	0.809
GCTA model(α=-1)	T3	0258	0.717
α-model(α=-1.25)	T1	0.261	0.807
α-model(α=-1.25)	T2	0.258	0.808
α-model(α=-1.25)	T3	0.275	0.765
α-model(α=-0.75)	T1	0.261	0.797
α-model(α=-0.75)	T2	0.264	0.806
α-model(α=-0.75)	T3	0.220	0.662
α-model(α=-0.5)	T1	0.257	0.789
α-model(α=-0.5)	T2	0.263	0.802
α-model(α=-0.5)	T3	0.171	0.611
α-model(α=-0.25)	T1	0.251	0.782
α-model(α=-0.25)	T2	0.261	0.798
α-model(α=-0.25)	T3	0.129	0.571
α-model(α=0.25)	T1	0.240	0.770
α-model(α=0.25)	T2	0.255	0.790
α-model(α=0.25)	T3	0.083	0.522
LDAK model(α=-0.25，LD window=1kb)	T1	0.256	0.783
LDAK model(α=-0.25，LD window=1kb)	T2	0.266	0.799
LDAK model(α=-0.25，LD window=1kb)	T3	0.135	0.573
LDAK model(α=-0.25，LD window=5kb)	T1	0.259	0.786
LDAK model(α=-0.25，LD window=5kb)	T2	0.272	0.800
LDAK model(α=-0.25，LD window=5kb)	T3	0.148	0.580
LDAK model(α=-0.25，LD window=50kb)	T1	0.266	0.788
LDAK model(α=-0.25，LD window=50kb)	T2	0.276	0.803
LDAK model(α=-0.25，LD window=50kb)	T3	0.147	0.591
LDAK model(α=-0.25，LD window=100kb)	T1	0.258	0.789
LDAK model(α=-0.25，LD window=100kb)	T2	0.270	0.803
LDAK model(α=-0.25，LD window=100kb)	T3	0.133	0.597
LDAK model(α=-0.25，LD window=500kb)	T1	0.262	0.796
LDAK model(α=-0.25，LD window=500kb)	T2	0.273	0.808
LDAK model(α=-0.25，LD window=500kb)	T3	0.089	0.625
LDAK model(α=-0.25，LD window=5000kb)	T1	0.329	0.799
LDAK model(α=-0.25，LD window=5000kb)	T2	0.347	0.807
LDAK model(α=-0.25，LD window=5000kb)	T3	0.070	0.684
